# Colorectal Cancer Screening Programme in Spain: Results of Key Performance Indicators After Five Rounds (2000–2012)

**DOI:** 10.1038/srep19532

**Published:** 2016-01-20

**Authors:** Gemma Binefa, Montse Garcia, Núria Milà, Esteve Fernández, Francisco Rodríguez-Moranta, Núria Gonzalo, Llúcia Benito, Ana Clopés, Jordi Guardiola, Víctor Moreno

**Affiliations:** 1 Cancer Prevention and Control Programme, Catalan Institute of Oncology, IDIBELL, Hospitalet de Llobregat, Barcelona, Spain; 2 Consortium for Biomedical Research in Epidemiology and Public Health (CIBERESP), Spain; 3 Department of Clinical Sciences, University of Barcelona, Hospitalet de Llobregat, Barcelona, Spain; 4 Department of Gastroenterology, University Hospital of Bellvitge, IDIBELL, Hospitalet de Llobregat, Barcelona, Spain; 5 Department of Pharmacy, Catalan Institute of Oncology, IDIBELL, Hospitalet de Llobregat, Barcelona, Spain; 6 Department of Fundamental Care and Medical-Surgical Nursing, University of Barcelona, Hospitalet de Llobregat, Barcelona, Spain

## Abstract

Effective quality assurance is essential in any screening programme. This article provides a unique insight into key quality indicators of five rounds of the first population-based colorectal cancer screening programme implemented in Spain (2000–2012), providing the results according to the type of screening (prevalent or first screen and incident or subsequent screen) and test (guaiac or immunochemical). The total crude participation rate increased from 17.2% (11,011) in the first round to 35.9% (22,988) in the last one. Rescreening rate was very high (88.6% in the fifth round). Positivity rate was superior with the faecal immunochemical test (6.2%) than with the guaiac-based test (0.7%) (p < 0.0001) and detection rates were also better with the immunochemical test. The most significant rise in detection rate was observed for high risk adenoma in men (45.5 per 1,000 screened). Most cancers were diagnosed at an early stage (61.4%) and there was a statistically significant difference between those detected in first or subsequent screening (52.6% and 70.0% respectively; p = 0.024). The availability of these results substantially improves data comparisons and the exchange of experience between screening programmes.

Screening for early detection of colorectal cancer (CRC) and its premalignant precursors has consistently demonstrated efficacy in reducing disease-specific mortality^1^. The European Union proposed faecal occult blood testing (FOBT) as the standard for CRC screening^2^,^3^ at about the same time as evidence supporting CRC was reported in a Cochrane review^4^.

During the last years, organized CRC screening programmes have been increasingly adopted throughout Europe^5^ and the immunochemical test (FIT) has emerged as a good option for screening^6^.

Since screening programmes invite healthy people with no symptoms, it is crucial to achieve an effective quality assurance to guarantee that the benefits of screening (better survival and quality of life) outweigh the harms (false negative result, false positive, complications related to colonoscopy as perforation or lower gastrointestinal bleeding)^7^,^8^. To that effect, screening programmes have the responsibility to ensure that quality is optimized in all ways: high quality, safe procedure and a satisfactory experience.

The European Union has proposed Key Performance Indicators for quality assurance and to monitor and compare the results of the screening programmes^9^.

In Spain, some regions implement and manage CRC screening programmes^10^,^11^. In 2000, Catalonia was the first region to launch a population-based CRC screening programme using FOBT in Hospitalet de Llobregat, a city of about 256,000 inhabitants in the metropolitan area of Barcelona^12^.

The objective of this study was to evaluate the five rounds (2000–2012) of the CRC screening programme and to identify those quality indicators that require improvement.

## Methods

Five rounds have been performed in the Catalan CRC biennial screening programme, from February 2000 to November 2012. Despite an intended 2-year interval between rounds, delays occurred in the first two rounds due to programming and management constraints. The screening programme began as a pilot with continuous monitoring of results and strategic modifications to accomplish the time intervals and other quality indicators. One of the most important modifications was the use of a more advanced informatics system and the incorporation of personal dedicated exclusively to the screening programme. Data to assess the screening process was obtained from the screening programme registry, which is a specific informatic data system only available for professionals of the programme. This registry integrates data from different healthcare informatics systems but the information is not accessible to everyone.

### Population

The target population included about 65,000 men and women aged 50–69 who were residents in the screening area, Hospitalet de Llobregat, a municipality to the immediate southwest of Barcelona. L’Hospitalet is the second largest city in Catalonia and one of the most densely populated cities in the European Union. About 2.2% of the population are illiterate, 11.9% have no education and the 16.3% have elementary education^13^.

Demographic data on this population was gathered from the Central Registry of Insured (Primary Healthcare Information System)^14^. L’Hospitalet is divided into 12 Health Care Centres and each individual is uniquely allocated to a general practitioner in one of these centres.

### Exclusion criteria

Subjects who did not meet the inclusion criteria for CRC screening were excluded according to the following criteria: personal history of CRC or adenomas, familial CRC cancer, inflammatory bowel disease, colonoscopy in the previous 5 years or FOBT in the previous 2 years, terminal disease or severe disabling condition. Subjects with an invalid mailing address and removals from the screening area registry were also excluded because they could not be invited to screening.

### Invitation process

Eligible subjects were invited by a personal letter offering a test completely free. A brochure with information regarding CRC and the screening process was sent with the invitation letter. The invitation was sent sequentially along the 12 Health Care Centres. A reminder letter was sent out if no response was obtained after six weeks of the initial invitation.

### FOBT (screening test)

Guaiac-based faecal occult blood test (gFOBT) was the only test used in the first three rounds (Hema-screen^TM^, immunostics.inc). In the fourth round, the FIT (OC Sensorμ, Palex) was partially introduced and offered to 12,707 individuals to assess its feasibility and acceptability. The gFOBT was offered to the rest of the target population (50,227). According to the good results with FIT, the Catalan Cancer Strategy decided that this screening test would be used throughout Catalonia. So in the fifth round the FIT was the unique test used.

Participants with gFOBT collected two samples of faeces from each of three consecutive stool specimens with no dietary restriction. The possible results of the gFOBT were 1) negative: zero out of six positive spots; 2) positive: five or six positive spots in a first gFOBT or one to six positive spots after a weak positive gFOBT; 3) weak positive: blood detected in one to four spots. Those participants were asked to perform a second gFOBT in this case with dietary restriction and, if any spot was positive, a colonoscopy was offered without further testing. In contrast, when all six spots were negative on the second test, a third gFOBT was requested, also with dietary restriction, to confirm a negative result; 4) inconclusive: expired samples (negative result with more than 14 days between faeces collection and its analysis), lack of information in the kit (personal identification number, date of faeces collection), or any technical failure or improper performance of the test. In any of these cases, a new gFOBT was also requested.

Dietary restriction consisted in a diet without foods containing peroxidase activity, such as uncooked vegetables or red meat, three days before the collection of samples.

Participants with FIT collected only one sample of faeces from one bowel movement. The cut-off was 100 ng/mL (20 μg Hb/g faeces). The results were categorized as positive (≥100 ng/mL), negative (<100 ng/mL) and inconclusive (the same reasons as gFOBT).

Individuals with inconclusive or negative final results were informed by mail. If there was no medical contraindication, subjects with a positive result were referred for colonoscopy and were offered an appointment date by phone.

### Colonoscopy (confirmatory examination)

A preoperative evaluation was routinely required (haemostasis test and electrocardiogram for all individuals and a chest x-ray only for individuals older than 64 years or with a chronic disease). Colonoscopies were performed with sedation on an outpatient basis at the Endoscopy Units of the county Hospitals by expert endoscopists using a specific afternoon agenda. Four days prior to colonoscopy, patients were phoned to remind them about the appointment to give instructions for the bowel preparation which was provided by primary health centres, either polyethylene glycol or sodium phosphate. Non-split dosing was used.

For those who chose to perform colonoscopy in a private clinic out of our programme, a postage paid envelope was provided in order to receive a copy of the colonoscopy report.

### Follow-up of colonoscopy results

If no adenomatous polyps were found, subjects were recorded to be invited for screening again after 10 years (provided that they still meet the inclusion criteria).

Any detected polyp was described and removed when endoscopically possible and sent for pathology review. Number, size (mm), morphology (pedunculated, sessile or flat) and location (rectum, sigmoid, descending, transverse, ascending or caecum) were documented. The follow-up of these lesions was transferred to the general practitioner or to the gastroenterology service.

For incomplete colonoscopies, patients were offered a new attempt of colonoscopy or another diagnostic exploration. Subjects with cancer or polyps too large or complicated to be removed endoscopically were referred to surgery. Major immediate complications were also documented (perforation, post-polypectomy bleeding involving transfusion or hospitalisation of at least 24 hours, and death within 30 days).

### Neoplasm classification

The histological classification of polyps and cancer was based on World Health Organization criteria^15^. Low-risk adenomas (LRA) were 1 or 2 lesions smaller than 10 mm, showing a tubular histology and low-grade dysplasia. High-risk adenomas (HRA) were defined as either adenomatous polyps ≥10 mm, or more than 2 adenomas, any adenoma with a tubulovillous or villous histology, or high-grade dysplasia. Carcinoma *in-situ* was classified as HRA. Advanced neoplasm included invasive carcinoma or HRA. All detected cases of invasive carcinoma were referred to the multidisciplinary CRC committee for appropriate oncology treatment. Tumours were staged according to the TNM system^16^.

During the study period, the European Guideline for Quality Assurance in Colorectal Cancer Screening and Diagnosis^9^ was published incorporating the intermediate risk adenoma (IRA) as a new category. Before its publication IRA was included into HRA. For the purpose of this study, we have maintained the initial risk classification. Nevertheless, recommendations for surveillance colonoscopies were made according to the most updated guidelines.

### Key Performance Indicators

In order to monitor the programme, we developed a series of key performance indicators (KPIs), including organizational (structural), process, and impact (short term outcomes) indicators (Table 1).

KPIs were analyzed by sex, age group and type of screening (prevalent or first screen and incident or subsequent screen) and type of screening test (gFOBT or FIT).

KPIs were compared with standard values published in the European Guideline^9^. The Catalan Advisory Group for Cancer Screening^17^ also edited a guideline following the principles of the European Guidelines. This latter added a new indicator (rescreening) and changed the standard value of 2 KPIs (time to colonoscopy and early-stage cancers). Definitions and measures of KPIs are shown in the Table 2.

We created a rating of each KPI according to its result: *“outstanding”* when the indicator exceeded 10% the reference value; *“acceptable”* when the indicator exceeded up to 10% of the standard, and *“needs for improvement”*, when the indicator was below the standard.

### Statistical methods

Rates and proportions were compared with Chi-square tests and logistic regression models. Odds ratios (OR) and 95% confidence intervals (CI) were calculated to estimate the differences. A p-value ≤ 0.05 was considered statistically significant.

### Ethics

Our CRC screening programme, like all Spanish population-based screening programmes, follows the Public Health laws and the Organic Law on Data Protection. The screening programme accomplishes the specific protocol based on the existing guidelines; the protocol was approved by the Ethics Committee of the University Hospital of Bellvitge. Moreover, informed consent was obtained from all the participants who underwent a colonoscopy.

## Results

Throughout five rounds, 114,559 people were invited, 37,953 participated at least once and 86,320 FOBT were performed. The KPIs of the five screening rounds are shown in Table 3.

### Organizational indicators

The coverage invitation rate was almost 100% in the five rounds. The programme invited the entire eligible population. In less than 1.5% the letter did not arrive due to errors in the address.

**Figure 1:**
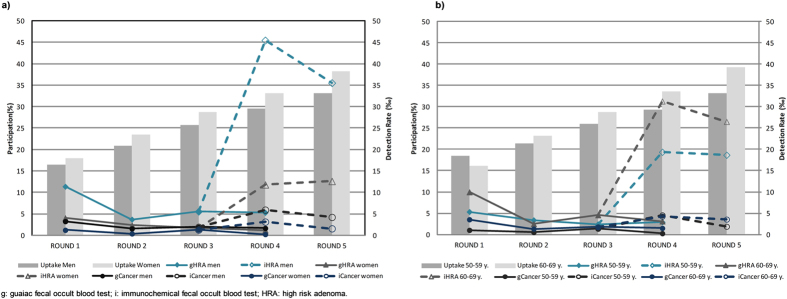
Participation and Detection Rates by sex (a) and age group (b).

The total crude participation rate increased from 17.2% in the first round to 35.9% in the last one which, though represents a statistically significant increment (p < 0.0001), was still far from the standard minimum desirable value (45%). Participation rate was consistently higher in women than men (p < 0.001) and in those between 60 and 69 years old, except for the first round (Fig. 1). Rescreening rate exceeded 80.0% in all rounds.

The standard of time to FOBT result was not achieved in the third round and time to colonoscopy did not reached the standard value in any round. Particular emphasis should be placed on FIT with only 36.4% of colonoscopies managed in the correct time interval in the fourth round, but reaching 61.7% in the fifth.

### Process indicators

Positivity rate was superior with FIT than gFOBT (p < 0.0001). Independently of the test used, subsequent screened had lower positivity rates than 1^st^ screened with the exception of FIT users in the 4^th^ round, where first and subsequent screen had the same positivity rate.

In all rounds the positivity rate was higher in the oldest group (60–69 years) and significantly higher in men than in women (p < 0.05).

Inconclusive FOBT rate decreased significantly in every round mainly in subsequent screenees (Table 3). The most common reason for coding an inconclusive FOBT was the incomplete information provided with the kit.

More than 95% of positive FOBT were referred for colonoscopy. The main reason for not recommending the exploration was having had a recent one. The majority of people accepted the colonoscopy as a diagnostic exploration. Therefore, colonoscopy compliance was one of the best process indicators, above the standard value (>85%) in all rounds.

The caecal intubation rate was within the range set by the European guideline as desirable. The most common reason for not reaching the caecum was stenosis.

The complication rate remained fairly constant at around 10 per 1,000 over time, except in the second round where no serious complications were reported, and in the fifth round, where the lowest rate was obtained (8.7 per 1,000). Seventy-nine percent of complications were post-polypectomy bleeding.

The Positive Predictive Value (PPV) for HRA was higher in the first screening compared to subsequent screening, except in the fifth round. PPV for cancer was also superior in the first screening except in the fourth round regardless of the test used (Table 3). The numbers, however, are small and no significant differences were observed.

Table 4 shows the results by type of test used independently of the round. We can observe that the PPV for advanced neoplasia (HRA or cancer) was higher in men than in women in all cases. When only gFOBT was used, the PPV for HRA and cancer were better in subsequent screening, except the PPV for HRA in women. Contrary, when FIT was the unique test used, the PPV was higher in first screening both in men and women. The results in the combined group can be considered similar to those obtained for FIT in first screenings.

### Impact indicators

An increase in detection rate was noticed with FIT. Both HRA and cancer detection rates in men and women were higher in first screening compared to subsequent screening (Table 4). The detection rate was much higher with FIT than with gFOBT and always higher in men than in women. In men, the advanced neoplasm detection rate in first screening with gFOBT and FIT was 10.7 and 48.0 per 1,000, respectively. Subsequent screening detection rates in men were 5.1 per 1,000 using gFOBT and 14.2 per 1,000 with FIT.

As shown in Fig. 1, the most significant rise in detection rate was observed for HRA in men who used FIT (45.5 per 1,000 screened) and in people aged more than 60 years old (31.3 per 1,000 screened). Cancer detection rate also showed an increase but less than HRA.

Overall, 61.4% of cancers were diagnosed at an early stage, with a statistically significant difference between those detected in first or subsequent screening (52.6% and 70.0%, respectively; p = 0.024).

Taking into account the results of the last round, most indicators reached or exceeded the acceptable standard value (Table 1).

## Discussion

This article provides a unique insight into KPI of the first five rounds of the earliest population-based CRC screening programme implemented in Spain. At the beginning, the results were poor for nearly all indicators. In the last round, only 4 indicators did not meet the European standards^9^ (participation, positivity, time to colonoscopy and initial stage cancers).

Participation rates in other Europeans CRC screening programmes with more than one round were higher than ours. The English, Scottish and French programmes reached a participation over 50% in their first round^18^,^19^,^20^. Surprisingly, in both the UK and France the participation fell in the 2nd round, which the authors attributed to a greater publicity in the first round when the piloting was launched. In other Spanish programmes, the participation rate was also greater than in ours^21^,^22^. In Hospitalet de Llobregat participation in the first round was only 17.2% but has increased progressively until 35.9% in the fifth round. Despite this poor participation, never reaching the standard of >45%^9^, it is noteworthy that the rescreening rate was outstanding (88.6% in the fifth round), which could suggest that participants are highly satisfied and accept the screening programme. Albeit participation rate when FIT was used was higher than with gFOBT (36.3% vs 30.2%), the rise was within the expected limits.

Like the vast majority of programmes, participation was higher among women than men. This sex gap in participation rates widened over time, being in the final round 37.7% and 33.9% respectively (p < 0.001).

As expected, the elderly were more likely to accept screening than younger population, except in the first round, and as in the case of sex, the difference increased along rounds.

We believe that the low participation rate reflects a lack of information of the complete screening process. Massive diffusion campaigns are needed to inform the general population about the high frequency of CRC in our setting, its primary risk factors and prevention measures, and the risks and benefits of taking part in screening programmes. The low participation could also be due to the high percentage of people with no education or primary education, comparing with Barcelona (the biggest Catalonian city), which has better participation rates but also lower illiteracy rates^12^,^23^. Finally, another point to consider for increasing participation rates is the migrant population living in the territory. Creating multilingual materials could be an additional strategy in areas with high migration rates.

FIT has many advantages with respect to gFOBT. Only one stool sample is necessary, no dietary restrictions are needed, the method for collecting is easier and less disgusting, it is possible to establish the most appropriate threshold level and, it has better sensitivity for detecting neoplasia^24^,^25^,^26^,^27^. On the other hand, the warm climate and the haemoglobin degradation over time may influence the FIT outcome, which would make it necessary to refrigerate the stool sample once it has been collected^28^,^29^.

The main reason for coding an inconclusive result for gFOBT and FIT was an improper performance of the test and the lack of the date of faeces collection, respectively. Our programme takes into account the date of collection of faeces due to the established relation with the higher degradation of haemoglobin with time^28^,^29^. So if a stool sample was collected more than fourteen days before its analysis and the result was negative, a new FOBT was required.

Positivity rate was the expected: higher positivity among men than in women and in the older group compared to the youngest. Positive rates with FIT were more than 7 times higher than gFOBT. Our results are in consonance with those published in the literature from screening programmes^16^,^30^ using the same FIT. Nevertheless, despite the forecast for the increase of diagnostic colonoscopies required by FIT, we were not able to carry them out within the appropriate time interval.

Colonoscopy compliance is a key indicator to consider when evaluating the effectiveness of the programme. In the first two rounds, despite acceptance being within the acceptable standard, it did not reach 90% as in following rounds. This might be due to the monitoring of people who had to undergo a colonoscopy done by the technical office. An average of 3 phone calls was made as a reminder of the appointment for the colonoscopy and the need of previous preparation.

More than 90% of colonoscopies reached the caecum. Caecal intubation is associated with sedation, since the participant’s welfare makes it possible to have more often an entire exploration and bowel preparation^31^. The need for completeness is based on the finding that an important number of CRC (near 30%) are located in the proximal colon. Caecal intubation rate is affected by a number of factors including bowel cleansing. Our adequate bowel cleansing rate was one of the highest reported in a screening programme^32^,^33^,^34^. We probably obtained this excellent result due to the extensive work done by the administrative staff who widely explained the whole process for a good bowel-cleansing and made a telephone reminder some days previous to the colonoscopy appointment. Inadequate bowel preparation not only limits the visibility of the mucosa and prolongs caecal intubation and withdrawal time, but it also leads to a shorter interval for the next exam. Nevertheless, the quality of bowel cleansing is a subjective measure and efforts to increase reproducibility and validity are needed^35^.

Complication rates should be interpreted carefully. The perforation rate considering the small number of colonoscopies performed, can largely vary from round to round and it is not considered a stable indicator. According to the European Guideline^9^, major complications (perforation and bleeding) occur in 0–3 per 1,000 colonoscopies in a high-quality CRC screening programme using colonoscopy as a primary screening test. On the other hand, the standard regarding major complications in people attending FOBT screening (as occurs in our programme) is 5–16 per 1,000 subjects undergoing colonoscopy. In that case the complication rate is higher because the colonoscopy is performed in individuals with a positive FOBT (screening test), which makes it more probable that they will have a neoplastic lesion and consequently more likely to have a complication derived from its removal.

Complication rate might be underestimated. We believe it is necessary to establish a system to detect complications after the patient has left the endoscopy department. Screening programmes are working to try to get as much information as possible, including minor complications that may arise before, during or after the colonoscopy.

The PPV improved with rounds, being clearly better in first than subsequent screenees. This indicator is closely linked to the false positive rate. False-positive tests lead to discomfort, costs and risks from additional diagnostic and therapeutic procedures. So it is important to identify factors associated with false-positive results. In our previous work we found that the risk of a false positive result increased with successive screening participations (OR = 1.72; 95% CI:1.20–2.46)^36^. Potential interference of drug intake such as antiplatelet and anticoagulation therapy and non-steroidal anti-inflammatory drugs has been reported, as well as the presence of haemorrhoids, with even contradictory results^37^,^38^,^39^,^40^.

Consistent with previous results^26^,^41^,^42^,^43^,^44^, FIT obtained much better detection rates than gFOBT. HRA detection rate never reached the standard value in any round with gFOBT. The introduction of FIT improved the detection rate, even among people who had previously participated with gFOBT.

Few programmes show the results according to first or subsequent screening. This is an important variable because the results vary depending on whether the individual participates for the first time or has been screened previously. Steele *et al.*^45^ observed that repeated invitations have a positive effect on participation in both groups They found acceptable neoplastic detection rates but, unlike our programme, they found worse cancer stage in incidence screenees.

Our programme, additionally, is one that has used both tests. The results of the group that participated with both tests can be useful for those programmes that are thinking to change its screening test from gFOBT to FIT and have information to the impact that it would suppose (especially the number of additional colonoscopies and detection rates).

Most cancers were diagnosed at an early stage, but this indicator did not reach the 70% target marked by the Catalan Advisory Group for Cancer Screening^17^. It is important to note that, in some CRC screening programmes, carcinomas *in situ* were included in the cancer group, which may increase the reported rates of early stage cancers. In our programme, carcinomas *in situ* were not considered cancers, but adenomas, with the consequent reduction in the reported rates of early stage cancer.

We consider that achieving a high detection rate for pre-neoplastic lesions is even more relevant than diagnosing early stage cancers. Detecting and removing preneoplastic lesions, stops the progression to cancer, therefore reduces the incidence of CRC and its associated mortality, increases survival and improves the quality of life.

Interval cancers should be recorded as a part of the screening process. The HRA detection rate has emerged as a potential measure of colonoscopy performance quality that correlates with interval cancer^46^. Interval cancer is a difficult indicator to measure, because of the need for adequate follow-up and a link between endoscopy and pathology reports, or cancer registry databases (which are unavailable in many countries as in our region)^33^,^47^.

The unknown completeness and accuracy of the central registry of insured could be a limitation. Nevertheless this registry was only used to get the target population and therefore, it was only involved in the coverage indicator. All other indicators were obtained from the specific colorectal cancer screening data base, which underwent to constant quality controls by the technical office.

Besides enhancing the results of the worst indicators, it is essential to maintain at good degree those with an acceptable result. The European guide is a first step for having quality standards of CRC screening programmes and allows comparisons between programmes or within the same programme across time, based on the same criteria. The availability of these standards substantially improves data comparisons and the exchange of experience between screening programmes in Europe and elsewhere. Nevertheless, further editions should incorporate new indicators such as those related with the adverse effects of the screening process as well as related costs.

In conclusion, the programme has identified as a priority to improve participation and to reduce the time between a positive FOBT result and the colonoscopy. Our results might point out the need to enhance awareness of screening among men (who have the lowest participation rate but the highest detection rates) and people who are screened for the first time and overcome participation barriers^48^.

It is also essential to carefully plan the agendas of colonoscopies needed to achieve the activity within the proper interval. The extension of the programme throughout Catalonia will be a reality soon and we must be prepared.

## Additional Information

**How to cite this article**: Binefa, G. *et al.* Colorectal Cancer Screening Programme in Spain: Results of Key Performance Indicators After Five Rounds (2000–2012). *Sci. Rep.* **6**, 19532; doi: 10.1038/srep19532 (2016).

## Figures and Tables

**Table 1:**
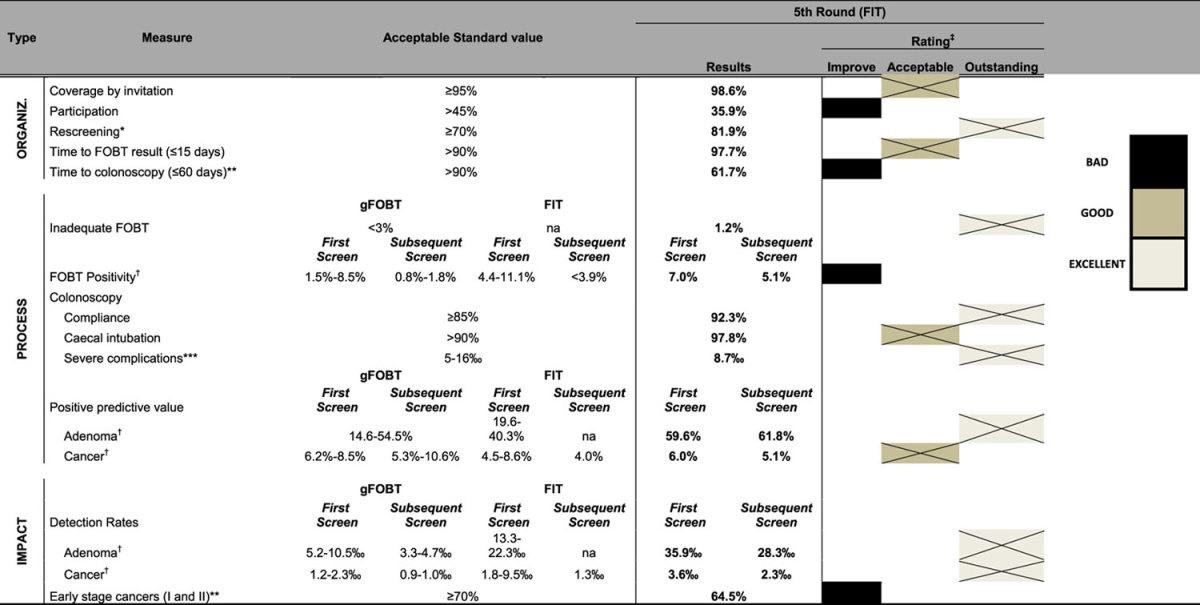
Acceptable standard value of the key performance indicators for colorectal cancer screening and results of the fifth round.

Organiz: Organizational. gFOBT: guaiac faecal occult blood test; FIT: faecal immunochemical test. ^‡^Improvement: results below the standard value; Acceptable: results up to 10% the standard value; Outstanding: ≥10% standard value. *This indicator is not included in the first edition of the European guidelines for quality assurance in colorectal cancer screening and diagnosis, 2011. **Acceptable standard value considered by the Catalan Advisory Group for Cancer Screening for the time interval to colonoscopy is >90% of colonoscopies performed within 60 days (instead of 31) and for the early stage cancers is ≥70% (instead of favourable). ***Data obtained from randomized controlled trials. ^†^Data obtained from population-based programmes. na: not data available.

**Table 2:** Colorectal cancer screening measures and indicators*.

Type	Indicator	Screening measure
ORGANIZATIONAL	Coverage rate by invitation (%) = A2/A1	A1 = Eligible.
		Total number of people eligible for screening according to the programme policy.
		A2 = Invited.
		Total number of people who received an invitation for screening according to the programme policy.
	Participation (%) = B/A2	B = Tested.
		Total number of people who have used and returned a FOBT kit irrespective of result. This includes people with inadequate/incomplete results. Note that each person is counted once regardless of the number of tests performed.
	Rescreening (%) = B/A3	A3 = Invited & previous screened.
		Total number of people invited for screening who participated in a previous round.
	Time to FOBT outcome (days) = C2-C1	C1 = Date of completion of FOBT.
		C2 = Date of receipt of results by the subject.
		To calculate this time interval we have used indirect measures. We used the date of the first stool sample and the date of sending the letter about the screening results.
	Time to colonoscopy (days) = D2-D1	D1 = Date of positive FOBT result.
		D2 = Date of colonoscopy after positive screening.
		We used the date recoded by the laboratory after analysing the FOBT and the date when the colonoscopy was performed.
PROCESS	Inadequate FOBT (%) = E2/B	E1 = Adequately tested.
		Total number of people who have returned a FOBT and achieved a conclusive result (positive or negative).
		E2 = Inadequately tested.
		Total number of people who have returned an inadequate FOBT (spoilt kit/technical failure or weak positive) and did not achieve a conclusive result.
	FOBT Positivity (%) = F/E1	F = Positive FOBT.
		Total number of people who have a positive/abnormal result with FOBT.
	Referral to colonoscopy (%) = G/F	G = Referred to colonoscopy.
		Total number of people presenting with a positive FOBT and referred for colonoscopy.
	Colonoscopy Compliance (%) = H/G	H = Diagnostic/therapeutic colonoscopy.
		Total number of people who have undergone a colonoscopy, including those whose colonoscopy was inadequate/ incomplete. Note that each person is counted once regardless of the number of colonoscopies which were performed.
	Caecal intubation (%) = K/ H	K = Caecal intubation (completion).
		Total number of complete colonoscopies (complete intubation of the colon and to carefully inspect the mucosa during withdrawal).
	Colonoscopic complications (‰) = L/H	L = Severe complications requiring hospitalizations.
		Total number of severe complications such as hospitalisation within 30 days due to serious haemorrhage involving transfusion, or due to perforation, vagal syndrome or peritonitis-like syndrome as a consequence of follow-up colonoscopy after positive screening.
	PPV LRA (%) =M1/H	M1 = LRA.
		Total number of people whose pathological specimens removed at endoscopy or surgery have been reported by a pathologist to be smaller lesions (≤10 mm) showing a tubular histology and low-grade dysplasia from all the people with colonoscopy performed.
	PPV HRA (%) = M2/H	M2 = HRA.
		Total number of people whose pathological specimens removed at endoscopy or surgery have been reported by a pathologist to be either adenomatous polyps larger or equal than 10mm, or more than 2 adenomas, any adenoma with a tubolovillous or villous histology, or high-grade dysplasia, from all the people with colonoscopy performed.
	PPV Cancer (%) = N/H	N = Cancers.
		Total number of people diagnosed with colorectal cancer by or as a direct result of the screening programme, from all the people with colonoscopy performed.
IMPACT	LRA detection rate (‰)= M1/E1	Total number of people whose pathological specimens removed at endoscopy or surgery have been reported by a pathologist to be smaller lesions (≤10 mm) showing a tubular histology and low-grade dysplasia from all the people with a FOBT adequately performed.
	HRA detection rate (‰)= M2/E1	Total number of people whose pathological specimens removed at endoscopy or surgery have been reported by a pathologist to be either adenomatous polyps larger or equal than 10mm, or more than 2 adenomas, any adenoma with a tubolovillous or villous histology, or high-grade dysplasia, from all the people with a FOBT adequately performed.
	Cancer detection rate (‰) = N/E1	Total number of people diagnosed with colorectal cancer by or as a direct result of the screening programme, from all the people with a FOBT adequately performed.
	Early- stage cancers (%) = O/N	O =Early- stage cancers (I and II).
		Total number of screen-detected cancers that were staged as I-II using the international TNM classification.

*Based on the European Guidelines for Quality Assurance in Colorectal Cancer Screening and Diagnosis – First Edition.

FOBT: faecal occult blood test; LRA: low risk adenoma; HRA: high risk adenoma.

**Table 3:** Results of the Colorectal Cancer Screening Key Performance Indicators per round.

	1st ROUND		2nd ROUND				3rd ROUND				4th ROUND								5th ROUND				
	gFOBT		gFOBT				gFOBT				gFOBT								FIT				
	n	(%)		n		(%)		n		(%)		n		(%)		n		(%)		n		(%)	
ORGANIZATIONAL																							
Coverage by Invitation	63,880	(99.6)		66,534		(99.8)		65,142		(99.6)		50,227		(99.6)		12,707		(99.8)		64,117		(98.6)	
Participation	11,011	(17.2)		14,818		(22.3)		17,742		(27.2)		15,143		(30.2)		4,618		(36.3)		22,988		(35.9)	
Rescreening	*			7,424		(73.2)		10,415		(87.0)		10,382		(87.3)		3,210		(89.7)		14,623		(88.6)	
Time to FOBT result (%≤15days)	**			**				16,652		(84.7)		14,360		(89.1)		3,938		(97.2)		21,581		(97.7)	
Time to Colonoscopy (%≤60days)	252	(75.4)		77		(72.0)		127		(70,9)		83		(88,3)		96		(36.4)		696		(61.7)	
PROCESS																							
Colonoscopy																							
Referral to Colonoscopy	372	(100.0)		123		(100.0)		182		(96.3)		99		(96.1)		280		(97.6)		1,238		(95.5)	
Compliance	334	(89.8)		108		(87.8)		180		(98.9)		96		(97.0)		271		(96.8)		1,147		(92.3)	
Caecal intubation	308	(92.2)		100		(92.6)		164		(91.1)		93		(96.9)		255		(94.1)		1,122		(97.8)	
Severe Complications	3	9.0‰		0		0.0‰		2		11.1‰		1		10.5‰		3		11.1‰		10		8.7‰	
		**1**^**st**^ **Scr.**		**1**^**st**^ **Scr.**		**Subs.**		**1**^**st**^ **Scr.**		**Subs.**		**1**^**st**^ **Scr.**		**Subs.**		**1**^**st**^ **Scr.**		**Subs.**		**1**^**st**^ **Scr.**		**Subs.**	
	**n**	**(%)**	**n**	**(%)**	**n**	**(%)**	**n**	**(%)**	**n**	**(%)**	**n**	**(%)**	**n**	**(%)**	**n**	**(%)**	**n**	**(%)**	**n**	**(%)**	**n**	**(%)**	
Inconclusive FOBT	383	(3.5)	360	(4.9)	276	(3.7)	265	(4.1)	301	(2.7)	92	(2.1)	107	(1.0)	32	(2.5)	18	(0.5)	219	(2.9)	62	(0.4)	
Positivity	372	(3.5)	79	(1.1)	44	(0.6)	74	(1.2)	115	(1.1)	39	(0.9)	64	(0.6)	78	(6.3)	209	(6.3)	509	(7.0)	788	(5.1)	
Positive Predictive Value																							
Low Risk Adenoma	22	(6.6)	5	(7.1)	2	(5.3)	4	(5.6)	9	(8.3)	2	(5.6)	4	(6.7)	7	(9.3)	17	(8.7)	65	(14.9)	119	(16.8)	
High Risk Adenoma	79	(23.7)	33	(47.1)	9	(23.7)	31	(43.7)	28	(25.7)	18	(50.0)	27	(45.0)	38	(50.7)	84	(42.9)	195	(44.6)	320	(45.1)	
Cancer	23	(6.9)	9	(12.9)	4	(10.5)	12	(16.9)	15	(13.8)	4	(11.1)	9	(15.0)	4	(5.3)	16	(8.2)	27	(6.2)	36	(5.1)	
IMPACT																							
Detection Rate		(‰)		(‰)		(‰)		(‰)		(‰)		(‰)		(‰)		(‰)		(‰)		(‰)		(‰)	
Low Risk Adenoma	22	(2.1)	5	(0.7)	2	(0.3)	4	(0.6)	9	(0.8)	2	(0.5)	4	(0.4)	7	(5.6)	17	(5.1)	65	(8.7)	119	(7.7)	
High Risk Adenoma	79	(7.4)	33	(4.7)	9	(1.3)	31	(5.0)	28	(2.6)	18	(4.3)	27	(2.5)	38	(30.5)	84	(25.3)	195	(26.1)	320	(20.6)	
Cancer	23	(2.2)	9	(1.3)	4	(0.6)	12	(1.9)	15	(1.4)	4	(1.0)	9	(0.8)	4	(3.2)	16	(4.8)	27	(3.6)	36	(2.3)	
		**(%)**		**(%)**		**(%)**		**(%)**		**(%)**		**(%)**		**(%)**		**(%)**		**(%)**		**(%)**		**(%)**	
Early Stage Cancers	13	(56.5)	6	(66.7)	3	(75.0)	5	(41.7)	9	(60.0)	1	(25.0)	7	(77.8)	2	(50.0)	11	(64.7)	15	(55.6)	26	(72.2)	

gFOBT: guaiac faecal occult blood test; FIT: immunochemical faecal occult blood test. *not applied; **not available. Scr: screen; Subs: subsequent.

**Table 4:** Comparisons of Positive Predictive Values and Detection Rates according to the test used during the screening period.

Tested	gFOBT									gFOBT+FIT			FIT								
	First			Subsequent			Overall			Subsequent			First			Subsequent			Overall		
		n			n			n			n			n			n			n	
Male		13,261			13,238			**26,499**			7,797			3,959			352			**4,311**	
Female		15,940			16,275			**32,215**			10,223			4,793			482			**5,275**	
		**29,201**			**29,513**			**58,714**			**18,020**			**8,752**			**834**			**9,586**	
																					
Positive FOBT & Diagnostic Endoscopy		**n**		**n**	**n**			**n**			**n**			**n**			**n**			**n**	
Male		278			113			**391**			508			316			16			**332**	
Female		233			94			**327**			368			196			14			**210**	
		**511**			**207**			**718**			**876**			**512**			**30**			**542**	
Endoscopy Result	**n**	**PPV**	**DR**	**n**	**PPV**	**DR**	**n**	**PPV**	**DR**	**n**	**PPV**	**DR**	**n**	**PPV**	**DR**	**n**	**PPV**	**DR**	**n**	**PPV**	**DR**
Male																					
Normal & polyps	120	43.2%	9.0‰	36	31.9%	2.7‰	156	39.9%	5.9‰	119	23.4%	15.3‰	76	24.1%	19.2‰	7	43.8%	19.9‰	83	25.0%	19.3‰
Low Risk Adenomas	16	5.8%	1.2‰	9	8.0%	0.7‰	25	6.4%	0.9‰	86	16.9%	11.0‰	49	15.5%	12.4‰	4	25.0%	11.4‰	53	16.0%	12.3‰
High Risk Adenomas	111	39.9%	8.4‰	47	41.6%	3.6‰	158	40.4%	6.0‰	273	53.7%	35.0‰	166	52.5%	41.9‰	5	31.3%	14.2‰	171	51.5%	39.7‰
Cancer	31	11.2%	2.3‰	21	18.6%	1.6‰	52	13.3%	2.0‰	30	5.9%	3.8‰	25	7.9%	6.3‰	0	0.0%	0.0‰	25	7.5%	5.8‰
Advanced Neoplasm	142	51.1%	10.7‰	68	60.2%	5.1‰	210	53.7%	7.9‰	303	59.6%	38.9‰	191	60.4%	48.2‰	5	31.3%	14.2‰	196	59.0%	45.5‰
Female																					
Normal	149	63.9%	9.3‰	64	68.1%	3.9‰	213	65.1%	6.6‰	181	49.2%	17.7‰	100	51.0%	20.9‰	7	50.0%	14.5‰	107	51.0%	20.3‰
Low Risk Adenomas	17	7.3%	1.1‰	6	6.4%	0.4‰	23	7.0%	0.7‰	41	11.1%	4.0‰	23	11.7%	4.8‰	6	42.9%	12.4‰	29	13.8%	5.5‰
High Risk Adenomas	50	21.5%	3.1‰	17	18.1%	1.0‰	67	20.5%	2.1‰	124	33.7%	12.1‰	67	34.2%	14.0‰	1	7.1%	2.1‰	68	32.4%	12.9‰
Cancer	17	7.3%	1.1‰	7	7.4%	0.4‰	24	7.3%	0.7‰	22	6.0%	2.2‰	6	3.1%	1.3‰	0	0.0%	0.0‰	6	2.9%	1.1‰
Advanced Neoplasm	67	28.8%	4.2‰	24	25.5%	1.5‰	91	27.8%	2.8‰	146	39.7%	14.3‰	73	37.2%	15.2‰	1	7.1%	2.1‰	74	35.2%	14.0‰

Tested: each person is counted once for each test performed. PPV: Positive Predictive Value; DR: Detection Rate → Endoscopy result/Screened population.
